# Drug-drug interactions of NOACs with comedication: Prevalence and implications for adverse events in patients with atrial fibrillation

**DOI:** 10.1016/j.hroo.2026.06.007

**Published:** 2026-06-18

**Authors:** Elisa Hennings, Stefanie Aeschbacher, Pia Neuschwander, Michael Coslovsky, Ioanna Istampoulouoglou, Anne Leuppi-Taegtmeyer, Rebecca E. Paladini, Lukas Strobel, Tobias Reichlin, Nicolas Rodondi, Jürg H. Beer, Giulio Conte, Angelo Auricchio, Giorgio Moschovitis, Marcello Di Valentino, Luise Adam, Maria Luisa De Perna, Leo H. Bonati, Felix Mahfoud, Philipp Krisai, Christine S. Zuern, David Conen, Christian Sticherling, Stefan Osswald, Michael Kühne, Swiss-AF Investigators

**Affiliations:** 1 Cardiovascular Research Institute Basel, University Hospital Basel, University of Basel, Basel, Switzerland; 2 Cardiology, University Hospital Basel, University of Basel, Basel, Switzerland; 3 Department of Clinical Research, University Hospital Basel, University of Basel, Basel, Switzerland; 4 Department of Clinical Pharmacology & Toxicology, University Hospital Basel, University of Basel, Basel, Switzerland; 5 Department of Patient Safety, Medical Directorate, University Hospital Basel, Basel, Switzerland; 6 Department of Cardiology, Inselspital, Bern University Hospital, University of Bern, Bern, Switzerland; 7 Department of General Internal Medicine, Inselspital, Bern University Hospital, University of Bern, Bern, Switzerland; 8 Institute of Primary Health Care (BIHAM), University of Bern, Bern, Switzerland; 9 Department of Internal Medicine, Cantonal Hospital Baden, Baden, Switzerland; 10 Molecular Cardiology, University Hospital of Zurich, Zurich, Switzerland; 11 Cardiology Division, Cardiocentro Ticino Institute, Regional Hospital of Lugano, Ente Ospedaliero Cantonale (EOC), Lugano, Switzerland; 12 Cardiology Division, Cardiocentro Ticino Institute, Regional Hospital of Lugano, Ente Ospedaliero Cantonale (EOC), Bellinzona, Switzerland; 13 Department of Neurology and Stroke Center, University Hospital Basel, University of Basel, Basel, Switzerland; 14 Research Department, Reha Rheinfelden, Rheinfelden, Switzerland; 15 Population Health Research Institute, McMaster University, Hamilton, Canada

**Keywords:** Atrial fibrillation, NOAC, Drug-drug interaction, Bleeding, Stroke

## Abstract

**Background:**

The clinical relevance of drug-drug interactions associated with non‒vitamin K-antagonist oral anticoagulants (NOACs) in patients with atrial fibrillation (AF) remains poorly understood.

**Objective:**

We evaluated the prevalence of pharmacokinetic and pharmacodynamic drug-drug interactions and their association with adverse clinical outcomes in patients with AF receiving NOACs.

**Methods:**

We analyzed patients from the prospective, multicenter Swiss-AF cohort study. We used frailty survival models to investigate the association of the different potential interactions with the respective clinical adverse outcomes (major bleeding, clinically relevant non-major bleeding, any bleeding, ischemic stroke or systemic embolism).

**Results:**

Our analysis comprised 1732 patients with AF (mean age 73 ± 8 years, 501 [29%] women) with a median follow-up period of 6 years. For 683 of these patients (39%), we recorded ≥1 potential drug-drug interaction. In the multivariable adjusted model, the hazard ratio of drug-drug interaction was 1.21 (95% confidence interval [CI] 0.84–1.75, *P* = .31) for major bleeding, 1.22 (95% CI 0.92–1.62, *P* = .17) for clinically relevant nonmajor bleeding, 1.22 (95% CI 0.97–1.54, *P* = .09) for any bleeding, and 1.00 (95% CI 0.22–4.53, *P* = .99) for ischemic stroke or systemic embolism.

**Conclusion:**

In a large real-world cohort of patients with AF, the prevalence of potential interactions of long-term comedication with NOACs was high. We found no strong evidence of an association between these interactions and an increased risk of bleeding or the composite outcome ischemic stroke or systemic embolism.

**Registration:**

Clinical Trial Registration: https://clinicaltrials.gov/ct2/show/NCT02105844

**ClinicalTrials.gov Identifier:**

NCT02105844

## Graphical abstract

**Figure:**
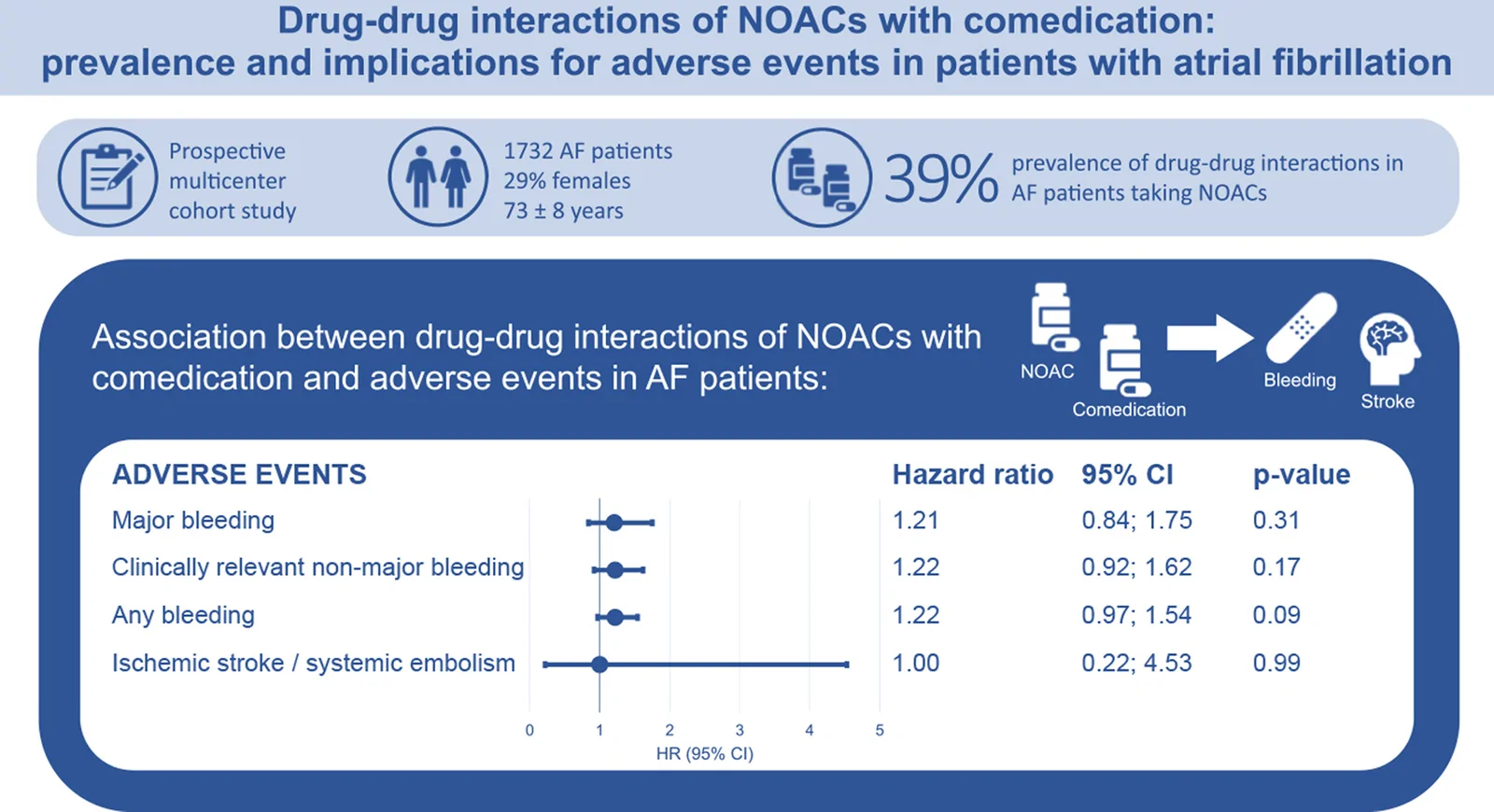

**Key Findings**

▪ In a large real-world cohort of patients with atrial fibrillation, the prevalence of potential drug–drug interactions between non‒vitamin K-antagonist oral anticoagulants and concomitant medications was high.
▪ Drug-drug interactions predominantly comprised low-level interactions.
▪ There was no strong evidence of an association between drug–drug interactions and an increased risk of bleeding or the composite outcome ischemic stroke or systemic embolism.

## Introduction

Patients with atrial fibrillation (AF) face a high risk of thromboembolic events such as stroke and systemic embolism.^1^ Non‒vitamin K-antagonist oral anticoagulants (NOACs) have emerged as a pivotal therapeutic cornerstone to reduce the risk of those events, but also increase the risk of bleeding.^1^ Moreover, they are not exempt from potential drug-drug interactions under simultaneous taking, which is particularly concerning in patients with AF who have a high burden of comorbidities and often require long-term treatment with multiple comedications. The pharmacokinetics of NOACs are largely determined by P-glycoprotein (P-gp) transporter and cytochrome P450 (CYP) enzymes.^2^ Concomitant medication may affect both the pharmacokinetics and pharmacodynamics of NOACs, potentially compromising their therapeutic plasma levels.^3^, ^4^, ^5^ This raises concerns about an increased risk of adverse events such as bleeding, stroke, and systemic embolism in these patients.

Because the clinical relevance of drug-drug interactions in patients with AF receiving NOACs remains poorly understood, we determined the prevalence of potential pharmacokinetic and pharmacodynamic drug-drug interactions in a large population of patients with AF receiving NOACs and evaluated the association between such drug-drug interactions and the adverse clinical outcomes bleeding and ischemic stroke or systemic embolism.

## Methods

### Study design, setting, and participants

We included patients from the ongoing prospective, multicenter Swiss Atrial Fibrillation (Swiss-AF) cohort study. Detailed methods have been previously published (ClinicalTrials.gov identifier: NCT02105844).^6^ In brief, Swiss-AF included 2415 patients with AF from 14 study sites in Switzerland recruited between 2014 and 2017. Inclusion criteria were age ≥65 years and documented AF by 12-lead electrocardiogram (ECG), or ≥30 seconds on a rhythm strip (eg, Holter ECG, implantable devices such as pacemaker, implantable cardioverter-defibrillator, loop recorders). A limited number of younger patients aged 45‒64 years were also included to investigate socioeconomic aspects in the working population. The exclusion criteria were the inability to sign informed consent, reversible forms of AF (eg, AF after cardiac surgery or severe sepsis), or acute illness within the last 4 weeks (eg, any hospitalization, acute infection, acute decompensated heart failure). The latter group of patients could be enrolled once the acute episode had resolved. We identified eligible patients by systematic screening in- and outpatients at participating study sites. Screening involved review of electronic health records and ECGs to identify patients meeting the inclusion criteria. For this analysis, we included patients from the Swiss-AF cohort who were receiving NOAC therapy at any time during the study period and had ≥1 follow-up visit. Continuous NOAC use throughout the entire follow-up was not required. We obtained written informed consent from each study participant. The study complied with the Declaration of Helsinki and was approved by local ethics committees at the participating sites (lead ethics committee: Ethikkommission Nordwest- und Zentralschweiz, Switzerland).

### Study variables

We used standardized questionnaires completed by study personnel to obtain data on the patients’ medical history, medication, and lifestyle at the baseline visit. Medical history was based on review of medical records, including hospital and outpatient reports, and was complemented by patient-reported information. The CHA_2_DS_2_-VASc score was calculated (Congestive heart failure, Hypertension, Age ≥ 75 years, Diabetes mellitus, Stroke, transient ischemic attack, or thromboembolism, Vascular disease, Age 65‒74 years, Sex category). We classified the type of AF into paroxysmal, persistent, and permanent according to the 2010 AF guidelines of the European Society of Cardiology, which were the guidelines at the time of study design.^7^ We recorded body weight, height, and blood pressure measured with calibrated devices and calculated the body mass index (weight in kilograms divided by height in meters squared). We measured blood pressure 3 times in a supine position after 5 minutes of rest, and the mean of the 3 measurements was used for the analysis. To determine the heart rhythm at enrolment, a resting 16-lead ECG of 5 minutes’ duration was obtained, and the rhythm was categorized into sinus rhythm, AF, or other. Nonfasting venous blood samples were obtained to assess kidney function and calculate the estimated glomerular filtration rate.^8^

### Drug-drug interactions

We collected comprehensive data on all concomitant medications using medical record review and standardized questionnaires at baseline and at each annual follow-up visit, including start and stop dates as precisely as possible. All recorded medications were subsequently systematically screened for potential pharmacokinetic and pharmacodynamic drug-drug interactions with NOACs using the following sources: the European Heart Rhythm Association practical guide on the use of NOACs,^9^ the online web application EasyDOAC www.easydoac.de,^10^ the Swiss medicinal product information Compendium®,^11^ and the evidence-based drug referential content Lexicomp®^12^ in consultation with expert pharmacologists at the Department of Clinical Pharmacology & Toxicology, University Hospital Basel, Switzerland. Potential pharmacokinetic and pharmacodynamic drug-drug interactions were systematically categorized into those “potentially increasing plasma levels of NOAC and higher bleeding risk” or those “potentially reducing plasma levels of NOAC and decreased oral anticoagulation effect.” Comedications were further classified into “caution required,” “not recommended,” and “contraindicated” according to the expected interaction strength (mild, moderate, or strong). Caution required means that the medication should be carefully prescribed, especially in the case of polypharmacy or when ≥2 interacting drugs or additional bleeding risk factors are present; if used, close clinical monitoring is recommended. Not recommended means that concomitant use should generally be avoided; if used, careful monitoring is required. Contraindicated means that concomitant use is not advisable and should be avoided owing to a high risk of clinically relevant interactions. Medications listed in the previously mentioned sources but labeled as no relevant interaction were not considered in this analysis. In cases of discrepancies among sources, classification was determined in consultation with the pharmacologists, considering the available evidence.

### Outcome variables

For the present analysis, the outcome variables of interest were major bleeding, clinically relevant nonmajor bleeding (CRNMB), any bleeding (ie, a composite outcome of major bleeding and CRNMB), and the composite outcome ischemic stroke or systemic embolism. The detailed definitions of these outcome variables are listed in Supplemental Table 1.

We assessed adverse clinical outcomes at the annual follow-up examination conducted on occasion of in-person visits or by telephone interviews. Clinical events were identified based on patient report and medical records. All available information from the treating physician or hospital was collected in case of an event. Events were then independently adjudicated by 2 physicians using standardized case report forms. In case of disagreement, a third physician was involved for final decision-making. In patients who could not be reached for follow-up, information was sought from designated contact persons and primary care physicians.

### Statistical analyses

Baseline characteristics are presented overall and stratified by potential drug-drug interaction at study inclusion. Categorical variables are presented as numbers (percentages) and were compared using Chi^2^-tests. Continuous variables are presented as means ± standard deviation or median (interquartile range [IQR]) and were compared using analysis of variance or Kruskal-Wallis test if strongly skewed. Distribution of continuous variables was checked using quantile-quantile plots.

Prevalence, number, and duration of potential drug-drug interactions in patients with AF taking NOACs were calculated. To investigate the association between potential drug-drug interactions and observed adverse clinical outcomes, we used a shared gamma frailty Cox proportional hazard model, which is an extension of the Cox proportional hazard model that accounts for the clustering of the data on patient identification, as implemented in R’s ‘survival’ package.^13^ For the frailty model, we only used the time intervals in which patients were on a NOAC and at risk of having drug-drug interactions. The use of comedication with potential for drug-drug interactions was used as the primary predictor variable, classified as previously described and updated over time. For bleeding, we used drug-drug interactions potentially leading to increased plasma levels of NOAC and increased bleeding risk as predictor. For the composite outcome ischemic stroke or systemic embolism, we used drug-drug interactions potentially leading to reduced plasma levels of NOAC and decreased oral anticoagulation effect as predictor. For concluding an association, drug-drug interactions and the adverse clinical event had to occur at the same time point. We conducted additional analyses treating pharmacokinetic and pharmacodynamic drug-drug interactions as separate predictors.

Initial models were adjusted for age and sex. Multivariable models were additionally adjusted for a predefined set of risk factors, comprising body mass index, systolic blood pressure, active smoking, history of diabetes, coronary artery disease, arterial hypertension, heart failure, stroke, transient ischemic attack, and estimated glomerular filtration rate. The covariates were selected on the basis of clinical plausibility, expert knowledge, and data availability in our cohort. Results are presented as hazard ratio (HR) and 95% CI.

All presented P-values are 2-sided. Considering the exploratory nature of the analysis, we performed no correction for multiple testing and interpreted P-values as a continuous variable that adds to the evidence against the relevant null hypothesis. Because only a few patients had missing covariate values, we removed these from the multivariable-adjusted analysis. We used all data available by May 2023. All analyses were performed using R version 4.1.2 (2021-11-01, R Core Team, Vienna, Austria).

## Results

### Participants

Of the 2415 patients participating in the Swiss-AF study, we excluded 669 patients because they had never taken any NOACs and 14 patients because they had no follow-up visits. This yielded a total of 1732 patients with AF for this analysis.

**Figure 1:**
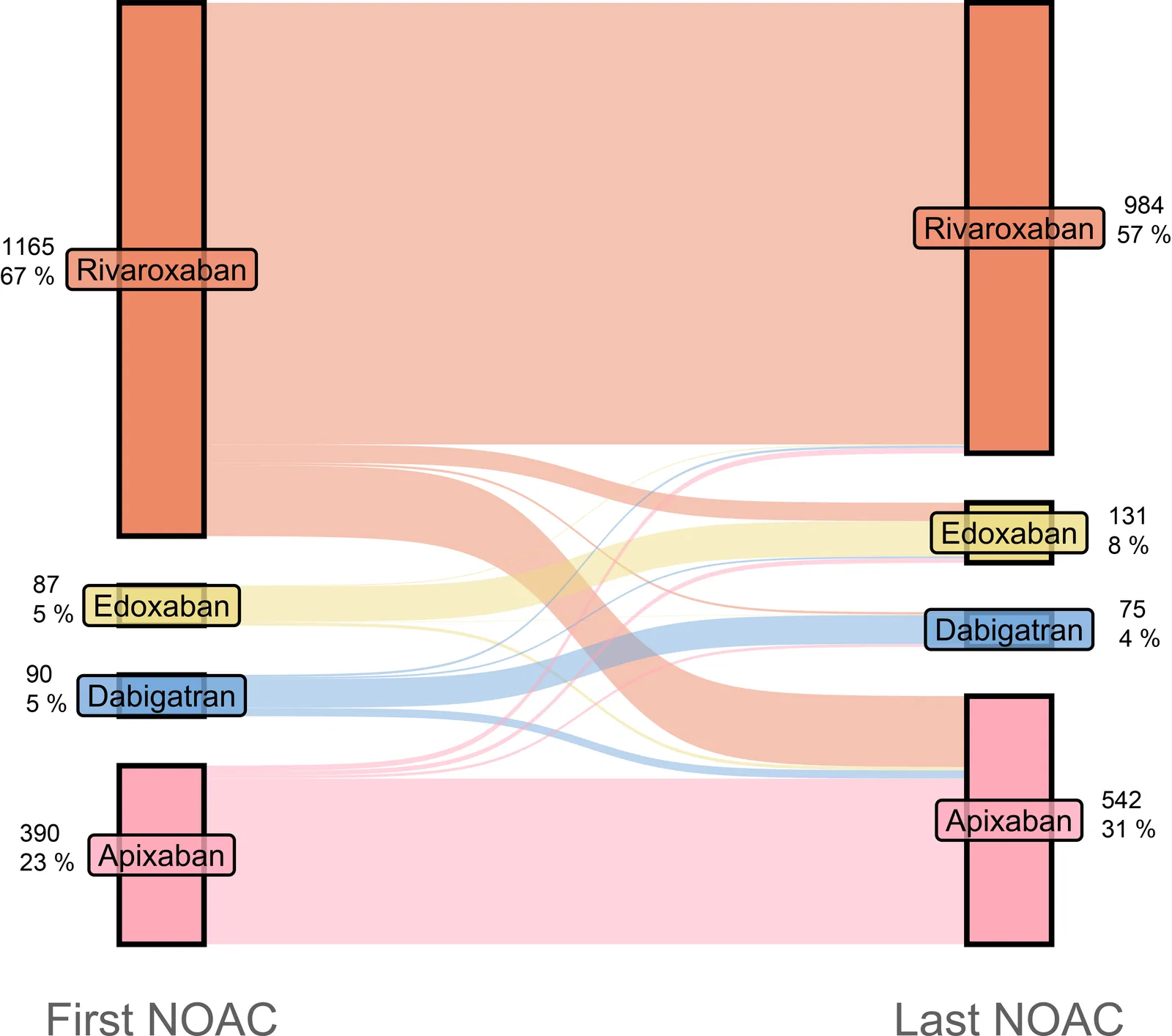
Distribution of the use of different NOACs in patients with atrial fibrillation. Sankey plot showing the number of patients with atrial fibrillation taking the different NOACs from the first to the last study visit. NOAC = non‒vitamin K-antagonist oral anticoagulant.

Table 1 lists the baseline characteristics overall and stratified by potential drug-drug interaction at study inclusion (baseline assessment). Mean age (± standard deviation) of the patients was 73 ± 8 years, and 501 of those (29%) were female. Patients with a potential drug-drug interaction were older than patients without interaction, had a higher CHA_2_DS_2_-VASc, and presented with diabetes less often but with coronary artery disease more often. The various types of NOACs (ie, apixaban, dabigatran, edoxaban, and rivaroxaban) were used by 390 (23%), 90 (5%), 87 (5%), and 1165 (67%) of the patients, respectively, with few of them changing the NOAC during the study (Figure 1).

**Table 1:** Baseline characteristics

*No. of patients*	Overall	Potential drug-drug interaction		No drug-drug interaction	*P*-value
		Potentially increased bleeding risk	Potentially reduced oral anticoagulation effect		
	n = 1732	n = 302	n = 9	n = 1421	
Age (y)	73 ± 8	74 ± 8	73 ± 5	72 ± 8	<.001
Female sex (n, %)	501 (29.0)	79 (26.2)	4 (44.4)	418 (29.4)	.31
Body mass index (kg/m^2^)	27.1 [24–30]	27 [25–31]	28 [26–30]	27 [24–30]	.47
Current smoker (n, %)	126 (7.3)	22 (7.3)	1 (11.1)	103 (7.3)	.91
Daily alcohol consumption (n, %)	1432 (82.8)	246 (81.5)	6 (66.7)	1180 (83.2)	.34
Heart rate (beats/min)	65 [58–75]	65 [58–75]	61 [58–82]	65 [58–75]	.91
Blood pressure (mmHg)					
Systolic	133 [121–146]	134 [124–147]	140 [129–161]	132 [121–146]	.16
Diastolic	78 [70–85]	75 [69–83]	76 [74–84]	78 [70–86]	.08
Atrial fibrillation type:					.87
Paroxysmal (n, %)	831 (48.0)	152 (50.3)	5 (55.6)	674 (47.4)	
Persistent (n, %)	567 (32.7)	94 (31.1)	3 (33.3)	470 (33.1)	
Permanent (n, %)	334 (19.3)	56 (18.5)	1 (11.1)	277 (19.5)	
Heart rhythm:					.55
Sinus rhythm (n, %)	885 (51.4)	149 (50.0)	6 (66.7)	730 (51.6)	
Atrial fibrillation (n, %)	557 (32.4)	104 (34.9)	3 (33.3)	450 (31.8)	
Other (n, %)	279 (16.2)	45 (15.1)	0	234 (16.5)	
CHA_2_DS_2_-VASc score	3 [2–5]	4 [3–5]	4 [2–5]	3 [2–4]	<.001
History of bleeding:					
Major bleeding (n, %)	95 (5.5)	14 (4.6)	2 (22.2)	79 (5.6)	.07
Clinically relevant nonmajor bleeding (n, %)	162 (9.4)	39 (12.9)	0	123 (8.7)	.05
History of coronary artery disease (n, %)	479 (27.7)	152 (50.3)	1 (11.1)	326 (22.9)	<.001
History of stroke or transient ischemic attack (n, %)	342 (19.8)	80 (26.5)	3 (33.3)	259 (18.2)	.003
History of systemic embolism (n, %)	81 (4.7)	15 (5.0)	1 (11.1)	65 (4.6)	.63
History of hypertension (n, %)	1221 (70.5)	230 (76.2)	6 (66.7)	985 (69.3)	.06
History of heart failure (n, %)	396 (22.9)	73 (24.2)	3 (33.3)	320 (22.5)	.62
History of diabetes mellitus (n, %)	278 (16.1)	63 (20.9)	3 (33.3)	212 (14.9)	.01
History of renal failure (n, %)	292 (16.9)	53 (17.6)	1 (11.1)	238 (16.8)	.84
History of peripheral artery disease (n, %)	128 (7.4)	30 (9.9)	1 (11.1)	97 (6.8)	.16
History of cancer (n, %)	263 (15.2)	53 (17.5)	0	210 (14.8)	.21
History of gastric ulcer (n, %)	75 (4.3)	16 (5.3)	0	59 (4.2)	.55
Recurrent falls (n, %)	143 (8.3)	28 (9.3)	1 (11.1)	114 (8)	.74
Laboratory values:					
eGFR (mL/min/1.7)	61 [50–73]	58 [47–73]	59 [52–74]	61 [50–73]	.14
Hemoglobin (g/L)	138 [126–150]	136 [122–147]	133 [115–143]	139 [126–151]	.04
Hematocrit (%)	41 [38–44]	40 [37–43]	39 [34–42]	41 [38–45]	.06
Thrombocytes (x10ˆ9/L)	216 [178–255]	206 [174–248]	230 [184–292]	218 [180–256]	.19

Values are mean ± standard deviation, median [interquartile range], or numbers (percentage). Baseline characteristics refer to the patients at study inclusion (not time-updated).

CHA_2_DS_2_-VASc = Congestive heart failure, Hypertension, Age ≥ 75 years (2 points), Diabetes mellitus, Stroke or transient ischemic attack or thromboembolism (2 points), Vascular disease, Age 65 to 74 years, Sex category female; eGFR = estimated glomerular filtration rate.

### Prevalence of drug-drug interactions

**Figure 2:**
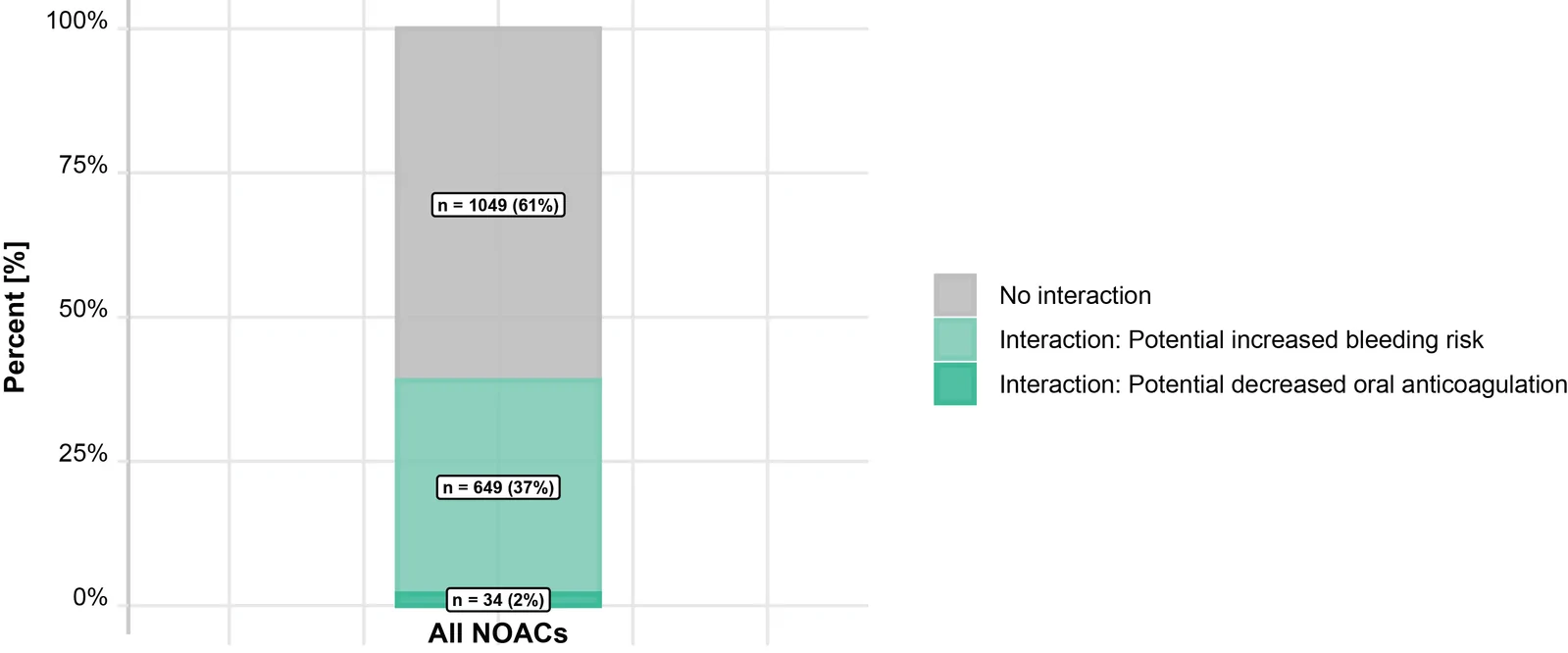
Prevalence of potential drug-drug interactions in patients with atrial fibrillation taking NOACs. Of 1732 patients with atrial fibrillation, 683 patients (39%) had ≥1 potential drug-drug interaction. Of these, 649 patients (37%) had potential drug-drug interactions possibly leading to increased plasma levels of NOAC and higher bleeding risk, and 34 patients (2%) had drug-drug interactions possibly leading to reduced plasma levels of NOAC and decreased oral anticoagulation effect. NOAC = non‒vitamin K-antagonist oral anticoagulant.

During a median follow-up period of 6 years (IQR 5–7), the recorded prevalence of drug-drug interactions with comedication in patients with AF receiving NOACs was 39% (37% with potentially increased bleeding risk, 2% with potentially decreased oral anticoagulation effect*;* Figure 2). The prevalence of potential drug-drug interactions per NOAC was 39% for apixaban, 54% for dabigatran, 42% for edoxaban, and 36% for rivaroxaban (Supplemental Figure 1).

**Figure 3:**
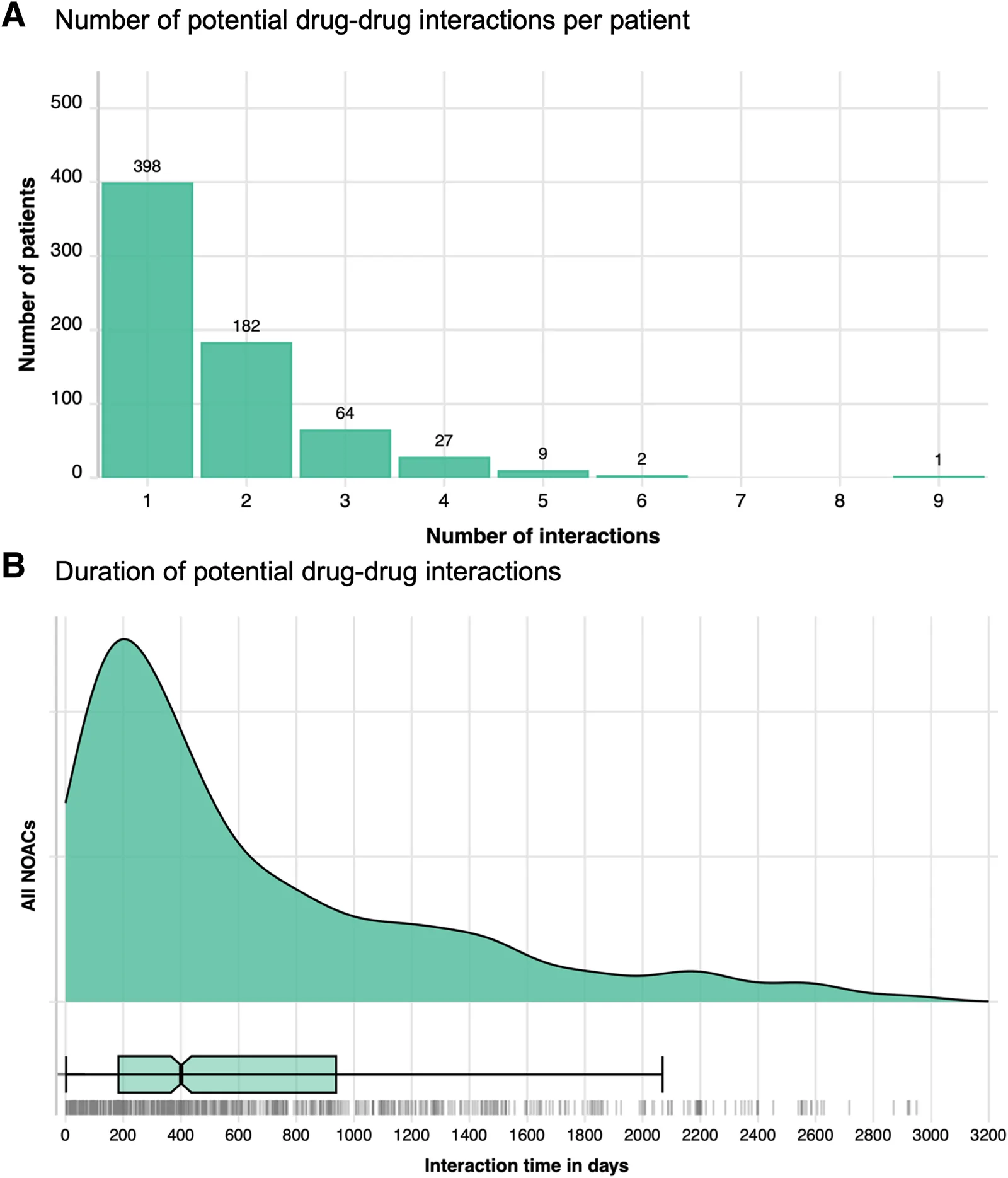
Number and duration of potential drug-drug interactions. **A:** Bar chart showing number of potential drug-drug interactions per patient. **B:** Ridgeline and box plot showing the duration (days) of potential drug-drug interactions. NOAC = non‒vitamin K-antagonist oral anticoagulant.

Overall, we identified a total of 1128 potential drug-drug interactions in 683 patients during the observed study period. Classification of labels comprised mostly caution required (1104 times, 97.9%), with not recommended (22 times, 2.0%) or contraindicated (2 times, 0.2%) stated in rare cases (Supplemental Table 2). The contraindicated category included 2 interactions involving bromelain with dabigatran and rivaroxaban. Not recommended interactions were predominantly observed with rivaroxaban and were primarily driven by interactions with dronedarone (potentially increasing bleeding risk) and with carbamazepine, enzalutamide, phenytoin, rifampicin, and St. John’s wort (potentially reducing anticoagulant effect) (Supplemental Table 3). The median number of potential drug-drug interactions per patient was 1 (IQR 1–2) for all NOACs combined (Figure 3A) as well as for the individual NOACs (Supplemental Figure 2), but a minority (103 patients [15.1%]) had ≥3 interactions. Median duration of drug-drug interactions was 401 days (IQR 184–938) (Figure 3B). Supplemental Figure 3 shows the duration of drug-drug interactions for the individual NOACs. Supplemental Table 4 lists the detailed drug-drug interactions listed by individual medication per individual NOAC, with the most common being platelet inhibitors, selective serotonin reuptake inhibitors (SSRI) or serotonin-norepinephrine reuptake inhibitors, nonsteroidal anti-inflammatory drugs (NSAIDs), ginkgo biloba, valerian, levetiracetam, and amiodarone; ie, pharmacodynamic drug-drug interactions were more frequent in our study cohort, whereas pharmacokinetic drug-drug interactions were less common.

### Association of potential drug-drug interactions with adverse clinical outcomes

**Figure 4:**
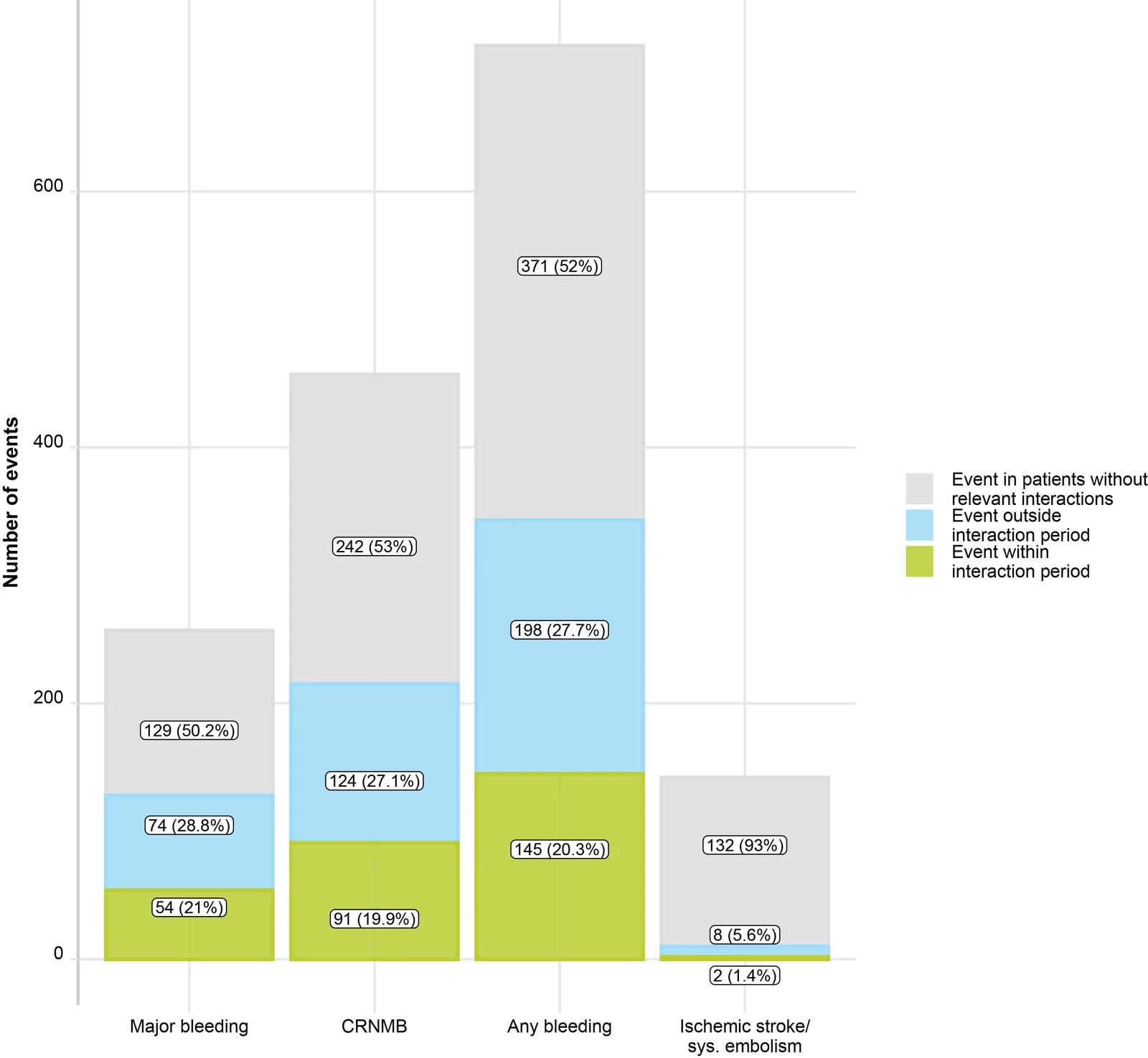
Number of adverse clinical outcomes occurring in patients without potential drug-drug interactions and number of adverse clinical outcomes occurring in patients with potential drug-drug interactions (outside an interaction period and within an interaction period). CRNMB = clinically relevant nonmajor bleeding; Sys. = systemic.

Figure 4 shows the numbers of adverse clinical outcomes occurring within or outside the interaction period. Supplemental Figure 4 shows the numbers of adverse clinical outcomes for the individual NOACs.

We identified 1073 potential drug-drug interactions in 649 patients, possibly leading to increased plasma levels of NOAC and higher bleeding risk. Major bleeding events were observed 257 times in 212 patients (incidence 2.06/100 person-years). From these, 128 events (49.8%) occurred in 101 patients with potential drug-drug interactions (54 events in 42 patients within a drug-drug interaction period, and 74 events in 65 patients outside interaction period) and 129 events (50.2%) in 111 patients without potential drug-drug interactions. CRNMB occurred 457 times in 336 patients (incidence 3.64/100 person-years), ie, 215 events (47.0%) in 153 patients with drug-drug interactions (91 events in 71 patients within a drug-drug interaction period, and 124 in 97 patients outside the interaction period) and 242 events (53.0%) in 183 patients without any potential drug-drug interactions. Bleeding of any type was observed 714 times in 477 patients (incidence 5.36/100 person-years), ie, 343 events (48.0%) in 219 patients with potential drug-drug interactions (145 times in 103 patients within a drug-drug interaction period, and 198 in 145 patients outside the interaction period), and 371 events (52.0%) in 258 patients without documented drug-drug interactions.

We identified 55 drug-drug interactions in 34 patients potentially leading to reduced plasma levels of NOACs and decreased oral anticoagulation effect. The composite outcome ischemic stroke or systemic embolism occurred 142 times in 121 patients (incidence 1.11/100 person-years), ie, 10 events (7.0%) in 9 patients with drug-drug interactions (2 events in 2 patients within drug-drug interaction period, 8 events in 7 patients outside the interaction period), and 132 events (93.0%) in 112 patients without drug-drug interactions. Numbers of events per NOAC according to the severity classification are listed in Supplemental Table 5.

**Figure 5:**
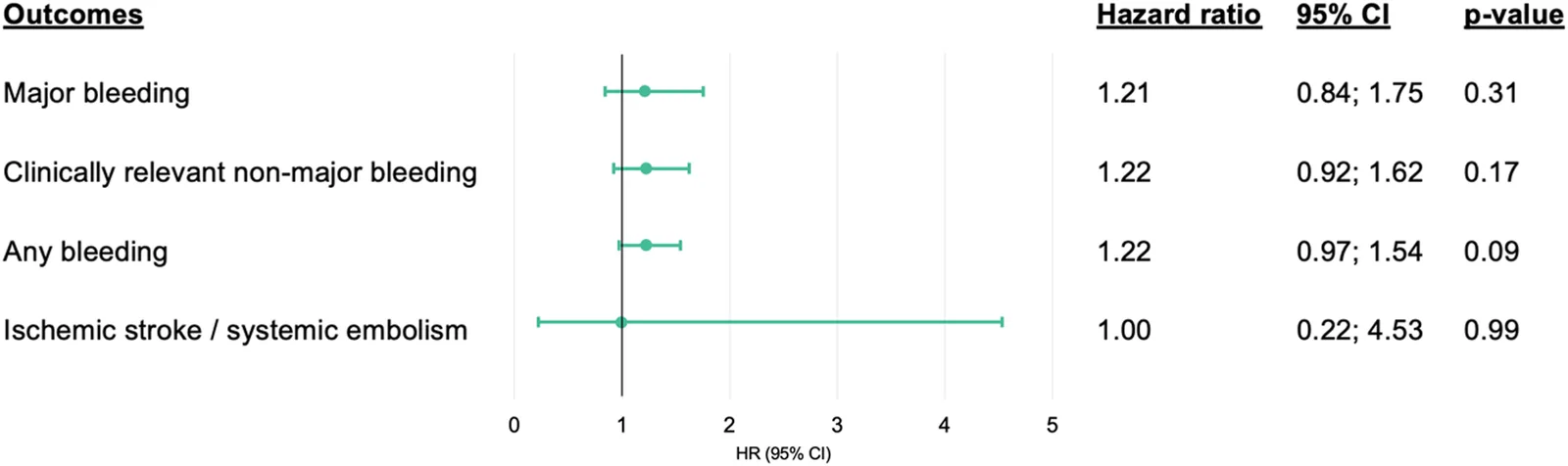
Association of potential drug-drug interactions with adverse clinical outcomes. HRs and 95% CIs from the multivariable-adjusted frailty models. CI = confidence interval; HR = hazard ratio.

Using the frailty model, the HR of potential drug-drug interaction in the model adjusted for age and sex was 1.29 (95% CI 0.90–1.85, *P* = .17) for major bleeding, 1.29 (95% CI 0.98–1.69, *P* = .06) for CRNMB, 1.28 (95% CI 1.03–1.61, *P* = .03) for any bleeding, and 1.16 (95% CI 0.24–5.36, *P* = .47) for ischemic stroke or systemic embolism. After multivariable adjustment, the HR was 1.21 (95% CI 0.84–1.75, *P* = .31) for major bleeding, 1.22 (95% CI 0.92–1.62, *P* = .17) for CRNMB, 1.22 (95% CI 0.97–1.54, *P* = .09) for any bleeding, and 1.00 (95% CI 0.22–4.53, *P* = .99) for ischemic stroke or systemic embolism (Figure 5). Results of the association between pharmacodynamic and pharmacokinetic drug-drug interactions and outcome events are comparable and presented in Supplemental Table 6.

## Discussion

Several important findings emerged from this comprehensive analysis of potential drug-drug interactions and adverse clinical outcomes in a large cohort of patients with AF receiving NOACs. First, the overall prevalence of potential drug-drug interactions of NOACs with long-term comedication was high but predominantly comprised low-level interactions. Second, drug-drug interactions potentially leading to an increased bleeding risk were particularly frequent, whereas those potentially leading to decreased anticoagulation effects were rare. Third, there was no strong association of drug-drug interactions with an increased risk for major bleeding or CRNMB. Fourth, we found no evidence of an increased risk for stroke or systemic embolism, but owing to the low prevalence, no definitive conclusions can be drawn. Finally, pharmacodynamic drug-drug interactions were more frequent than pharmacokinetic interactions, with the most common drugs being platelet inhibitors, SSRI or serotonin-norepinephrine reuptake inhibitors, NSAIDs, ginkgo biloba, valerian, levetiracetam, and amiodarone.

According to the European Society of Cardiology guidelines for the diagnosis and management of AF, NOACs are the first-line treatment choice to prevent stroke in patients with AF.^1^ In daily routine, NOACs have replaced vitamin K-antagonists (VKAs) in most indications except in certain cases such as in patients with mechanical prosthetic valve.^9^ Although at least as effective as VKAs in stroke prevention,^14^ NOACs offer several benefits over VKAs, such as faster onset and stop of action, a standardized dosing schedule without the need for regular therapeutic monitoring, reduced risk of intracranial bleeding, and fewer interactions with drugs and food.^3^^,^^9^^,^^14^ Nevertheless, owing to their pharmacokinetics and pharmacodynamics, NOACs are not devoid of potential drug-drug interactions, raising concerns about the impact on therapeutic plasma concentrations and subsequent risk of bleeding or stroke.^9^ Because polypharmacy in the patient population with multimorbid AF is very common (eg, polypharmacy in 64% of patients in the Rivaroxaban versus Warfarin in atrial fibrillation (ROCKET-AF) trial and 77% in the Apixaban versus Warfarin in atrial fibrillation (ARISTOTLE) trial,^15^^,^^16^ potential drug-drug interactions with comedications must be addressed in clinical routine.

The prevalence of potential drug-drug interactions with NOACs in our cohort of real-world patients with AF was 39%, which is consistent with the percentage reported in a Swedish population-based cohort study (approximately 50%).^17^ In our cohort, most of the potential drug-drug interactions alerted to drugs potentially leading to increased plasma levels of NOACs and increasing the bleeding risk (37%) on the basis of both pharmacokinetic and pharmacodynamic interactions. In contrast, drugs potentially leading to reduced plasma levels of NOACs and decreased oral anticoagulation effect, thus potentially increasing the risk of ischemic stroke or systemic embolism, were rare (2%).

Pharmacokinetic interactions are due to concomitant drugs influencing the plasma levels of NOACs. In particular, interaction mechanisms include the excretion of NOACs by the P-gp efflux transporters in addition to their metabolism by cytochrome P450 enzymes (ie, CYP3A4).^9^ P-gp efflux transporters are mostly found in the gastrointestinal tract (to a certain extent also in the liver and kidneys), and all 4 NOACs are excreted through these.^9^ Metabolism through CYP3A4 is relevant for hepatic clearance of apixaban and rivaroxaban.^9^ Consequently, competitive inhibition of P-gp efflux transporters or CYP3A4 enzyme by concomitant drugs may lead to increased plasma levels of NOACs, thus potentially increasing the bleeding risk in patients. This is particularly relevant for patients with AF because several drugs commonly used in this patient population are P-gp inhibitors (eg, verapamil, amiodarone) or CYP3A4 inhibitors (eg, clarithromycin). Conversely, potent inducers of the P-gp transporter and CYP3A4 enzyme (eg, rifampicin, carbamazepine, valproic acid, St. John’s wort) may reduce NOAC plasma levels, thus potentially decreasing the anticoagulant effect. In addition, certain medications interact with the pharmacodynamics of NOACs. These include drugs affecting hemostasis, thus also potentially increasing the risk of bleeding, eg, platelet inhibitors, NSAIDs, or SSRI.^4^

We analyzed the association of the potential drug-drug interactions with actual adverse clinical outcomes recorded in our study. Across all bleeding outcomes—major bleeding, CRNMB, and any bleeding—we did not observe a significantly increased risk in patients with drug-drug interactions. In the pharmacokinetic interaction subgroup, HRs for major bleeding were numerically elevated, although associations did not reach statistical significance and CIs were wide. Given this was an exploratory analysis without formal power analysis, our results should be interpreted with caution and in light of the available literature.

A meta-analysis based on 10 post hoc analyses of randomized controlled trials and 5 cohort studies analyzing patients with AF receiving NOACs and concomitant P-gp or CYP3A4 inhibitors found an increased risk of major bleeding (relative risk 1.10 [95% CI 1.01‒1.19]).^3^ This is not consistent with the effect we found in our cohort, which had a smaller sample size than the meta-analysis. We also included medications with pharmacodynamic effects in our study, which differed from the meta-analysis that specifically analyzed the effects of P-gp or CYP3A4 inhibitors, ie, pharmacokinetic interactions. However, it is noteworthy that the studies in this meta-analysis assessed drug-drug interactions at baseline, but 13 of 15 studies did not account for the initiation of novel drugs with interacting effects during follow-up, and 9 studies did not account for the discontinuation of them. Our analysis was based on time-updated drug-drug interactions, and the event had to occur at precisely the same time. Regarding pharmacodynamic drug-drug interactions only, 3 previous studies showed that concomitant use of NOACs is associated with a higher risk of bleeding.^4^^,^^17^^,^^18^

Only a few studies investigated P-gp and CYP3A4 inducers and their association with ischemic stroke, and they revealed conflicting results.^17^^,^^19^^,^^20^ One study was a retrospective population-based cohort study of data from national Swedish registries.^17^ Among 3314 patients receiving P-gp or CYP3A4 inducers, the authors did not find any evidence of an increased risk of ischemic stroke. A recent Belgian analysis of nationwide data from 2903 patients receiving P-gp or CYP3A4 inducers found a strong association of these drugs with the risk of stroke but not with the combined end point stroke or systemic embolism.^20^ The mean follow-up period was much shorter than in our study (mean of 0.8 years vs median of 6 years). An Israeli retrospective nested case-control study analyzing 383 patients found a strong association of P-gp and CYP3A4 inducers with the incidence of the composite outcome stroke and systemic embolism.^19^ In our cohort, we found no evidence of a strong association of drug-drug interactions with stroke or systemic embolism. However, owing to a low number of the relevant drug-drug interactions (potentially reduced plasma levels of NOAC and decreased oral anticoagulation effect) and a low number of stroke and systemic embolism events in our study, no definitive conclusions can be drawn. The analysis should therefore be considered exploratory, with limited power to detect meaningful associations.

An important consideration in interpreting our findings is that most of the identified drug-drug interactions were classified as low level (caution required = 97.9%), reflecting predominantly pharmacodynamic interactions rather than high-risk pharmacokinetic interactions directly affecting NOAC plasma levels. Consequently, our results primarily apply to a well-managed population in whom high-risk combinations were rarely observed. This likely reflects guideline-concordant prescribing and may explain the largely neutral associations with major clinical outcomes. Therefore, our findings should not be extrapolated to high-risk drug-drug interactions, which remain clinically relevant but were under-represented in this cohort.

### Strengths and limitations

Our study had several strengths. In our well-characterized cohort of patients with AF, we were able to adjust for important confounders. Our analysis was time-updated with respect to the type of comedications. Outcome events were systematically evaluated with standardized case report forms, and the events were validated by ≥2 physicians.

Our study also had certain limitations. We did not determine the actual blood levels of NOACs in patients with potential drug-drug interactions. In addition, medication exposure was assessed at annual study visits, capturing long-term comedication and not short-term use of interacting drugs. This could have caused misclassification of exposure and may have biased the results toward the null. In addition, information on prescription refills or objective measures of medication adherence were not available. Although loss to follow-up was low (n = 35), incomplete follow-up information in a small number of patients may have caused missed clinical events. Because our study was conducted in Switzerland and primarily includes Caucasians, the generalizability of our findings is limited. Lastly, owing to the observational study design, residual confounding might persist despite multivariable adjustment.

Future research should use more frequent medication assessments to capture episodic drug exposures such as short-course antibiotics. Furthermore, studies investigating whether therapeutic drug monitoring of NOAC plasma levels can guide dose adjustments in patients with drug-drug interactions may improve safety, although evidence-based therapeutic ranges are lacking.

From a clinical perspective, systematic regular drug-drug interaction screening including over-the-counter drugs and supplements should be implemented through electronic health record alerts, integrating not only drug-drug interactions but also other high-risk features for bleeding or stroke such as older age or renal impairment. Patients should also be counseled to avoid over-the-counter medication such as NSAIDs and St. John’s wort.

## Conclusion

Within our large real-world cohort of patients with AF, the prevalence of potential drug-drug interactions of NOACs with long-term comedication was high, but predominantly comprised low-level interactions. We found no strong evidence supporting an association between drug-drug interactions and an increased risk of bleeding or the composite outcome ischemic stroke or systemic embolism.

## Disclosures

Jürg H. Beer is supported by the Swiss National Foundation of Science 324730_182328, the Swiss Heart Foundation, the Kardio Foundation; he has received grant support and consultation fees to the institution from Bayer, Daiichi Sankyo, and Sanofi. Leo H. Bonati reports personal fees and nonfinancial support from Amgen, grants from AstraZeneca, personal fees and nonfinancial support from Bayer, personal fees from Bristol-Myers Squibb, personal fees from Claret Medical, grants from the Swiss National Science Foundation, grants from the University of Basel, and grants from the Swiss Heart Foundation, outside the submitted work. David Conen received consultation fees from Roche Diagnostics and Trimedics. Philipp Krisai reports research grants from the Swiss National Science Foundation, Swiss Heart Foundation, the Machaon Foundation, and the Foundation for Cardiovascular Research Basel, and speaking fees from BMS/Pfizer. Michael Kühne reports grants from the Swiss National Science Foundation (Grant numbers 33CS30_148474, 33CS30_177520, 32473B_176178, 32003B_197524, and 33IC30_229733), the Swiss Heart Foundation, the Foundation for Cardiovascular Research Basel and the University of Basel, grants from Abbott, Bayer, BMS, Boston Scientific, Daiichi Sankyo, Medtronic, Pfizer, and Roche, and royalties from Springer (ECG book). Felix Mahfoud is supported by Deutsche Gesellschaft für Kardiologie (DGK), Deutsche Forschungsgemeinschaft (SFB TRR219, Project-ID 322900939), and Deutsche Herzstiftung. Until May 2024, he received speaker honoraria/consulting fees from Ablative Solutions, Amgen, Astra-Zeneca, Bayer, Boehringer Ingelheim, Inari, Medtronic, Merck, ReCor Medical, Servier, and Terumo, all outside the submitted work. Giorgio Moschovitis has received advisory board or speaker’s fees from Astra Zeneca, Bayer, Boehringer Ingelheim, Daiichi Sankyo, Gebro Pharma, Novartis, and Vifor, all outside the submitted work. Stefan Osswald received research grants from the Swiss National Science Foundation and Swiss Heart Foundation, Foundation for CardioVascular Research Basel, and F. Hoffmann-La Roche Ltd, and educational and speaker grants from F. Hoffmann-La Roche Ltd, Bayer, Novartis, Sanofi, AstraZeneca, Daiichi-Sankyo, and Pfizer. Tobias Reichlin received research grants from the Swiss National Science Foundation, the Swiss Heart Foundation, the sitem insel support funds, Biotronik, Boston Scientific and Medtronic, all for work outside the submitted study; speaker/consulting honoraria or travel support from Abbott/SJM, Bayer, Biosense-Webster, Biotronik, Boston-Scientific, Farapulse, Medtronic, Pfizer-BMS, all for work outside the submitted study; and support for his institution’s fellowship program from Abbott/SJM, Biosense-Webster, Biotronik, Boston-Scientific, and Medtronic for work outside the submitted study. Christian Sticherling is a member of the Medtronic Advisory Board Europe and Boston Scientific Advisory Board Europe, received educational grants from Biosense Webster and Biotronik, a research grant from the European Union’s FP7 program, and Biosense Webster and lecture and consulting fees from Abbott, Medtronic, Biosense-Webster, Boston Scientific, Microport, and Biotronik all outside the submitted work.

The remaining authors declare no conflict of interest.

## Footnotes

Appendix Supplementary data associated with this article can be found in the online version at https://doi.org/10.1016/j.hroo.2026.06.007.
