# Supplementary material for: Drug-drug interactions of NOACs with comedication: Prevalence and implications for adverse events in patients with atrial fibrillation
Source: Heart Rhythm O2. 2026 Jun 18;7(9):1680–90. doi: 10.1016/j.hroo.2026.06.007 (PMC13594476; doi:10.1016/j.hroo.2026.06.007)
Supplement: Supplementary file 1 [file mmc1.docx]

Supplemental

Drug-drug interactions of NOACs with long-term medication: prevalence and implications for adverse events in patients with atrial fibrillation

Elisa Hennings MD PhD^1,2^*, Stefanie Aeschbacher PhD^1,2^*, Pia Neuschwander PhD^3^, Michael Coslovsky PhD^3^, Ioanna Istampoulouoglou MD^4^, Anne Leuppi-Taegtmeyer MD^3,5,^, Rebecca E. Paladini PhD^1,2^, Lukas Strobel^1,2^, Tobias Reichlin MD^6^, Nicolas Rodondi MD^7,8^, Jürg H. Beer MD^9,10^, Giulio Conte MD^11^, Angelo Auricchio MD^11^, Giorgio Moschovitis MD^11^, Marcello Di Valentino MD^12^, Luise Adam MD^8^, Maria Luisa De Perna MD^11^, Leo H. Bonati MD^13,14^, Felix Mahfoud MD^1,2^, Philipp Krisai MD^1,2^, Christine S. Zuern MD^1,2^, David Conen MD MPH^15^, Christian Sticherling MD^1,2^, Stefan Osswald MD^1,2^, Michael Kühne MD^1,2^, on behalf of the Swiss-AF Investigators^16^

1 Cardiovascular Research Institute Basel, University Hospital Basel, University of Basel, Basel, Switzerland
2 Cardiology, University Hospital Basel, University of Basel, Basel, Switzerland
3 Department of Clinical Research, University Hospital Basel, University of Basel, Basel, Switzerland
4 Department of Clinical Pharmacology & Toxicology, University Hospital Basel, University of Basel, Basel, Switzerland
5 Department of Patient Safety, Medical Directorate, University Hospital Basel, Basel, Switzerland
6 Department of Cardiology, Inselspital, Bern University Hospital, University of Bern, Bern, Switzerland
7 Department of General Internal Medicine, Inselspital, Bern University Hospital, University of Bern, Bern, Switzerland
8 Institute of Primary Health Care (BIHAM), University of Bern, Bern, Switzerland
9 Department of Internal Medicine, Cantonal Hospital Baden, Baden, Switzerland
10 Molecular Cardiology, University Hospital of Zurich, Zurich, Switzerland
11 Cardiology Division, Cardiocentro Ticino Institute, Regional Hospital of Lugano, Ente Ospedaliero Cantonale (EOC), Lugano, Switzerland
12 Cardiology Division, Cardiocentro Ticino Institute, Regional Hospital of Lugano, Ente Ospedaliero Cantonale (EOC), Bellinzona, Switzerland
13 Department of Neurology and Stroke Center, University Hospital Basel, University of Basel, Basel, Switzerland
14 Research Department, Reha Rheinfelden, Rheinfelden, Switzerland
15 Population Health Research Institute, McMaster University, Hamilton, Canada
16 All Swiss-AF Investigators are listed in the supplement

* equal contribution

| **Index** |  | Page |
| --- | --- | --- |
| **Figure S1** | Prevalence of potential drug-drug interactions in AF patients stratified by individual NOACs | 3 |
| **Figure S2** | Number of potential drug-drug interactions per patient stratified by individual NOACs | 3 |
| **Figure S3** | Duration of potential drug-drug interactions stratified by individual NOACs | 4 |
| **Figure S4** | Number of adverse events stratified by individual NOACs | 4 |
| **Table S1** | Definitions of outcome variables | 7 |
| **Table S2** | Potential drug-drug interactions listed by severity classification | 7 |
| **Table S3** | Potential drug-drug interactions listed by individual medication and severity classification stratified by NOAC group | 8 |
| **Table S4** | Potential drug-drug interactions listed by individual medication taken by the study participants | 9 |
| **Table S5** | Number of adverse events per NOAC according to severity classification | 10 |
| **Table S6** | Association of pharmacodynamic and pharmacokinetic drug–drug interactions with adverse outcome events | 11 |
| **Swiss-AF Investigators** | | 12 |

**Figure S1:** Prevalence of potential drug-drug interactions in AF patients stratified by individual NOACs

**Figure S2:** Number of potential drug-drug interactions per patient stratified by individual NOACs

**Figure S3:** Duration of potential drug-drug interactions stratified by individual NOACs

**Figure S4:** Number of adverse events stratified by individual NOACs

Number of adverse events occurring in patients without potential drug-drug interactions and adverse events occurring in patients with potential drug-drug interactions (outside an interaction period and within an interaction period).

(A) Major bleeding

(B) Clinically relevant non-major bleeding

(C) Any bleeding

(D) Ischemic stroke/systemic embolism

| **Table S1:** Definitions of outcome variables | |
| --- | --- |
| **Outcome variable** | **Definition** |
| Major bleeding | Major bleeding was defined according to the criteria of the International Society on Thrombosis and Haemostasis.^1^ Thus, major bleeding was clinically overt bleeding with a fatal outcome, reduction in hemoglobin level of ≥ 20 g/L within 7 days, transfusion of at least two units of blood, or symptomatic bleeding in a critical area or organ (i.e., intracranial, intraspinal, intraocular, pericardial, intra-articular, intramuscular with compartment syndrome, retroperitoneal).^1^ |
| Clinically relevant non-major bleeding | Clinically relevant non-major bleeding was defined as bleeding not fulfilling the criteria for major bleeding, but that was clinically overt and led to hospitalization, change of antithrombotic therapy, or medical or surgical intervention.^2^ |
| Ischemic stroke | Ischemic stroke was defined as a rapid onset of focal neurologic dysfunction with clinical, imaging, or pathologic evidence of focal infarction of the brain, retina (excluding anterior ischemic optic neuropathy), or spinal cord explaining the dysfunction. Clinical evidence of infarction was based on symptoms persisting ≥ 24 h or until death, and exclusion of other etiologies (i.e., brain infection, trauma, tumor, seizure, severe metabolic disease, degenerative neurologic disease). |
| Systemic embolism | Systemic embolism was assumed if there was clear evidence of abrupt occlusion of a systemic artery consistent with an embolic event. This included clinical signs and symptoms as well as one of the following: surgical report indicating evidence of arterial embolism, pathologic specimens related to embolism removal, imaging evidence consistent with arterial embolism, or autopsy reports. Pulmonary embolism or deep vein thrombosis were excluded from outcome variables. |

References: 1. Schulman S, Kearon C. Definition of major bleeding in clinical investigations of antihemostatic medicinal products in non-surgical patients. J Thromb Haemost. 2005;3:692–694. 2. Kaatz S, Ahmad D, Spyropoulos AC, Schulman S. Definition of clinically relevant non-major bleeding in studies of anticoagulants in atrial fibrillation and venous thromboembolic disease in non-surgical patients: communication from the SSC of the ISTH. J Thromb Haemost. 2015;13:2119–2126.

| **Table S2:** Potential drug-drug interactions listed by severity classification | | | | | | | | | | | | | | | |
| --- | --- | --- | --- | --- | --- | --- | --- | --- | --- | --- | --- | --- | --- | --- | --- |
|  |  | NOAC | | | | | | | | | | |  |  |  |
|  |  | Apixaban | |  | Dabigatran | |  | Edoxaban | |  | Rivaroxaban | |  |  |  |
| Classification | | N | % |  | N | % |  | N | % |  | N | % |  | Total | % |
| Potentially increased bleeding risk | Caution required | 294 | 93.04 |  | 82 | 92.13 |  | 84 | 96.55 |  | 603 | 94.81 |  | 1063 | 94.24 |
|  | Not recommended | 0 | 0 |  | 0 | 0 |  | 0 | 0 |  | 8 | 1.26 |  | 8 | 0.71 |
|  | Contraindicated | 0 | 0 |  | 1 | 1.12 |  | 0 | 0 |  | 1 | 0.16 |  | 2 | 0.18 |
| Potentially decreased oral anticoagulation effect | Caution required | 22 | 6.96 |  | 4 | 4.49 |  | 3 | 3.45 |  | 12 | 1.89 |  | 41 | 3.63 |
|  | Not recommended | 0 | 0 |  | 2 | 2.25 |  | 0 | 0 |  | 12 | 1.89 |  | 14 | 1.24 |
| All interactions |  | 316 | 100 |  | 89 | 100 |  | 87 | 100 |  | 636 | 100 |  | 1128 | 100 |

| **Table S3:** Potential drug-drug interactions listed by individual medication and severity classification stratified by NOAC group | | | | | | | | | | | | | | | |
| --- | --- | --- | --- | --- | --- | --- | --- | --- | --- | --- | --- | --- | --- | --- | --- |
|  |  | NOAC | | | | | | | | | | |  |  |  |
|  |  | Apixaban | |  | Dabigatran | |  | Edoxaban | |  | Rivaroxaban | |  |  |  |
| Medication | Classification | N | % |  | N | % |  | N | % |  | N | % |  | Total | % |
| Amiodarone | Caution required | 0 | 0 |  | 21 | 23.6 |  | 0 | 0 |  | 0 | 0 |  | 21 | 1.86 |
| Aspirin | Caution required | 94 | 29.75 |  | 19 | 21.35 |  | 29 | 33.33 |  | 210 | 33.02 |  | 352 | 31.21 |
| Bromelain | Contraindicated | 0 | 0 |  | 1 | 1.12 |  | 0 | 0 |  | 1 | 0.16 |  | 2 | 0.18 |
| Carbamazepine | Caution required | 3 | 0.95 |  | 0 | 0 |  | 1 | 1.15 |  | 0 | 0 |  | 4 | 0.36 |
|  | Not recommended | 0 | 0 |  | 0 | 0 |  | 0 | 0 |  | 3 | 0.47 |  | 3 | 0.27 |
| Carvedilol | Caution required | 0 | 0 |  | 4 | 4.49 |  | 0 | 0 |  | 0 | 0 |  | 4 | 0.36 |
| Ciclosporine | Caution required | 2 | 0.63 |  | 0 | 0 |  | 0 | 0 |  | 1 | 0.16 |  | 3 | 0.27 |
| Clopidogrel | Caution required | 47 | 14.87 |  | 6 | 6.74 |  | 10 | 11.49 |  | 85 | 13.36 |  | 148 | 13.12 |
| Curcumin | Caution required | 1 | 0.32 |  | 0 | 0 |  | 0 | 0 |  | 0 | 0 |  | 1 | 0.09 |
| Dronedarone | Caution required | 0 | 0 |  | 0 | 0 |  | 1 | 1.15 |  | 0 | 0 |  | 1 | 0.09 |
|  | Not recommended | 0 | 0 |  | 0 | 0 |  | 0 | 0 |  | 8 | 1.26 |  | 8 | 0.71 |
| Enzalutamide | Caution required | 3 | 0.95 |  | 0 | 0 |  | 2 | 2.3 |  | 0 | 0 |  | 5 | 0.44 |
|  | Not recommended | 0 | 0 |  | 0 | 0 |  | 0 | 0 |  | 2 | 0.31 |  | 2 | 0.18 |
| Ginkgo biloba | Caution required | 32 | 10.13 |  | 5 | 5.62 |  | 8 | 9.2 |  | 46 | 7.23 |  | 91 | 8.07 |
| Horse chestnut | Caution required | 0 | 0 |  | 0 | 0 |  | 0 | 0 |  | 1 | 0.16 |  | 1 | 0.09 |
| Hydroxychloroquine | Caution required | 0 | 0 |  | 1 | 1.12 |  | 0 | 0 |  | 0 | 0 |  | 1 | 0.09 |
| Levetiracetam | Caution required | 11 | 3.48 |  | 4 | 4.49 |  | 0 | 0 |  | 8 | 1.26 |  | 23 | 2.04 |
| Mirabegron | Caution required | 0 | 0 |  | 2 | 2.25 |  | 0 | 0 |  | 0 | 0 |  | 2 | 0.18 |
| Nilotinib | Caution required | 1 | 0.32 |  | 0 | 0 |  | 0 | 0 |  | 0 | 0 |  | 1 | 0.09 |
| Nonsteroidal anti-inflammatory drug (NSAID) | Caution required | 42 | 13.3 |  | 8 | 8.99 |  | 13 | 14.94 |  | 109 | 17.14 |  | 172 | 15.25 |
| Oxcarbazepine | Caution required | 1 | 0.32 |  | 0 | 0 |  | 0 | 0 |  | 1 | 0.16 |  | 2 | 0.18 |
| Phenytoin | Not recommended | 0 | 0 |  | 0 | 0 |  | 0 | 0 |  | 2 | 0.31 |  | 2 | 0.18 |
| Prasugrel | Caution required | 0 | 0 |  | 0 | 0 |  | 1 | 1.15 |  | 2 | 0.31 |  | 3 | 0.27 |
| Propafenone | Caution required | 0 | 0 |  | 2 | 2.25 |  | 1 | 1.15 |  | 0 | 0 |  | 3 | 0.27 |
| Ranolazine | Caution required | 0 | 0 |  | 0 | 0 |  | 2 | 2.3 |  | 0 | 0 |  | 2 | 0.18 |
| Rifampicin | Not recommended | 0 | 0 |  | 0 | 0 |  | 0 | 0 |  | 2 | 0.31 |  | 2 | 0.18 |
| Selective serotonin reuptake inhibitor (SSRI) | Caution required | 46 | 14.56 |  | 10 | 11.24 |  | 11 | 12.64 |  | 90 | 14.15 |  | 157 | 13.92 |
| Serotonin–norepinephrine reuptake inhibitor (SNRI) | Caution required | 19 | 6.01 |  | 2 | 2.25 |  | 6 | 6.9 |  | 33 | 5.19 |  | 60 | 5.32 |
| St. John’s wort | Caution required | 3 | 0.95 |  | 0 | 0 |  | 0 | 0 |  | 0 | 0 |  | 3 | 0.27 |
|  | Not recommended | 0 | 0 |  | 2 | 2.25 |  | 0 | 0 |  | 3 | 0.47 |  | 5 | 0.45 |
| Ticagrelor | Caution required | 0 | 0 |  | 0 | 0 |  | 0 | 0 |  | 1 | 0.16 |  | 1 | 0.09 |
| Valerian | Caution required | 10 | 3.16 |  | 1 | 1.12 |  | 0 | 0 |  | 25 | 3.93 |  | 36 | 3.19 |
| Valproic acid | Caution required | 1 | 0.32 |  | 0 | 0 |  | 0 | 0 |  | 3 | 0.47 |  | 4 | 0.36 |
| Verapamil | Caution required | 0 | 0 |  | 1 | 1.12 |  | 2 | 2.3 |  | 0 | 0 |  | 3 | 0.27 |
| All interactions |  | 316 | 100 |  | 89 | 100 |  | 87 | 100 |  | 636 | 100 |  | 1128 | 100 |

| **Table S4:** Potential drug-drug interactions listed by individual medication taken by the study participants | | | | | | | | | | | | | | |
| --- | --- | --- | --- | --- | --- | --- | --- | --- | --- | --- | --- | --- | --- | --- |
|  | NOAC | | | | | | | | | | |  |  |  |
|  | Apixaban | |  | Dabigatran | |  | Edoxaban | |  | Rivaroxaban | |  |  |  |
| Medication | N | % |  | N | % |  | N | % |  | N | % |  | Total | % |
| Amiodarone | 0 | 0 |  | 21 | 23.6 |  | 0 | 0 |  | 0 | 0 |  | 21 | 1.86 |
| Aspirin | 94 | 29.75 |  | 19 | 21.35 |  | 29 | 33.33 |  | 210 | 33.02 |  | 352 | 31.21 |
| Bromelain | 0 | 0 |  | 1 | 1.12 |  | 0 | 0 |  | 1 | 0.16 |  | 2 | 0.18 |
| Carbamazepine | 3 | 0.95 |  | 0 | 0 |  | 1 | 1.15 |  | 3 | 0.47 |  | 7 | 0.62 |
| Carvedilol | 0 | 0 |  | 4 | 4.49 |  | 0 | 0 |  | 0 | 0 |  | 4 | 0.36 |
| Ciclosporine | 2 | 0.63 |  | 0 | 0 |  | 0 | 0 |  | 1 | 0.16 |  | 3 | 0.27 |
| Clopidogrel | 47 | 14.87 |  | 6 | 6.74 |  | 10 | 11.49 |  | 85 | 13.36 |  | 148 | 13.12 |
| Curcumin | 1 | 0.32 |  | 0 | 0 |  | 0 | 0 |  | 0 | 0 |  | 1 | 0.09 |
| Dronedarone | 0 | 0 |  | 0 | 0 |  | 1 | 1.15 |  | 8 | 1.26 |  | 9 | 0.80 |
| Enzalutamide | 3 | 0.95 |  | 0 | 0 |  | 2 | 2.3 |  | 2 | 0.31 |  | 7 | 0.62 |
| Ginkgo biloba | 32 | 10.13 |  | 5 | 5.62 |  | 8 | 9.2 |  | 46 | 7.23 |  | 91 | 8.07 |
| Horse chestnut | 0 | 0 |  | 0 | 0 |  | 0 | 0 |  | 1 | 0.16 |  | 1 | 0.09 |
| Hydroxychloroquine | 0 | 0 |  | 1 | 1.12 |  | 0 | 0 |  | 0 | 0 |  | 1 | 0.09 |
| Levetiracetam | 11 | 3.48 |  | 4 | 4.49 |  | 0 | 0 |  | 8 | 1.26 |  | 23 | 2.04 |
| Mirabegron | 0 | 0 |  | 2 | 2.25 |  | 0 | 0 |  | 0 | 0 |  | 2 | 0.18 |
| Nilotinib | 1 | 0.32 |  | 0 | 0 |  | 0 | 0 |  | 0 | 0 |  | 1 | 0.09 |
| Nonsteroidal anti-inflammatory drug (NSAID) | 42 | 13.3 |  | 8 | 8.99 |  | 13 | 14.94 |  | 109 | 17.14 |  | 172 | 15.25 |
| Oxcarbazepine | 1 | 0.32 |  | 0 | 0 |  | 0 | 0 |  | 1 | 0.16 |  | 2 | 0.18 |
| Phenytoin | 0 | 0 |  | 0 | 0 |  | 0 | 0 |  | 2 | 0.31 |  | 2 | 0.18 |
| Prasugrel | 0 | 0 |  | 0 | 0 |  | 1 | 1.15 |  | 2 | 0.31 |  | 3 | 0.27 |
| Propafenone | 0 | 0 |  | 2 | 2.25 |  | 1 | 1.15 |  | 0 | 0 |  | 3 | 0.27 |
| Ranolazine | 0 | 0 |  | 0 | 0 |  | 2 | 2.3 |  | 0 | 0 |  | 2 | 0.18 |
| Rifampicin | 0 | 0 |  | 0 | 0 |  | 0 | 0 |  | 2 | 0.31 |  | 2 | 0.18 |
| Selective serotonin reuptake inhibitor (SSRI) | 46 | 14.56 |  | 10 | 11.24 |  | 11 | 12.64 |  | 90 | 14.15 |  | 157 | 13.92 |
| Serotonin–norepinephrine reuptake inhibitor (SNRI) | 19 | 6.01 |  | 2 | 2.25 |  | 6 | 6.9 |  | 33 | 5.19 |  | 60 | 5.32 |
| St. John’s wort | 3 | 0.95 |  | 2 | 2.25 |  | 0 | 0 |  | 3 | 0.47 |  | 8 | 0.71 |
| Ticagrelor | 0 | 0 |  | 0 | 0 |  | 0 | 0 |  | 1 | 0.16 |  | 1 | 0.09 |
| Valerian | 10 | 3.16 |  | 1 | 1.12 |  | 0 | 0 |  | 25 | 3.93 |  | 36 | 3.19 |
| Valproic acid | 1 | 0.32 |  | 0 | 0 |  | 0 | 0 |  | 3 | 0.47 |  | 4 | 0.36 |
| Verapamil | 0 | 0 |  | 1 | 1.12 |  | 2 | 2.3 |  | 0 | 0 |  | 3 | 0.27 |
| All interactions | 316 | 100 |  | 89 | 100 |  | 87 | 100 |  | 636 | 100 |  | 1128 | 100 |

| **Table S5:** Number of adverse events per NOAC according to severity classification | | | | | | | | | | | | | | | |
| --- | --- | --- | --- | --- | --- | --- | --- | --- | --- | --- | --- | --- | --- | --- | --- |
|  |  | NOAC | | | | | | | | | | |  |  |  |
| Adverse events | Classification | Apixaban | |  | Dabigatran | |  | Edoxaban | |  | Rivaroxaban | |  |  |  |
|  | | N |  |  | N |  |  | N |  |  | N |  |  | Total |  |
| Major bleeding | Caution required | 25 |  |  | 0 |  |  | 4 |  |  | 38 |  |  | 67 |  |
|  | Not recommended | 0 |  |  | 0 |  |  | 0 |  |  | 0 |  |  | 0 |  |
|  | Contraindicated | 0 |  |  | 0 |  |  | 0 |  |  | 0 |  |  | 0 |  |
| Clinically relevant non-major bleeding | Caution required | 27 |  |  | 7 |  |  | 12 |  |  | 66 |  |  | 112 |  |
|  | Not recommended | 0 |  |  | 0 |  |  | 0 |  |  | 0 |  |  | 0 |  |
|  | Contraindicated | 0 |  |  | 0 |  |  | 0 |  |  | 0 |  |  | 0 |  |
| Any bleeding | Caution required | 52 |  |  | 7 |  |  | 16 |  |  | 104 |  |  | 179 |  |
|  | Not recommended | 0 |  |  | 0 |  |  | 0 |  |  | 0 |  |  | 0 |  |
|  | Contraindicated | 0 |  |  | 0 |  |  | 0 |  |  | 0 |  |  | 0 |  |
| Ischemic stroke/systemic embolism | Caution required | 1 |  |  | 0 |  |  | 0 |  |  | 1 |  |  | 2 |  |
|  | Not recommended | 0 |  |  | 0 |  |  | 0 |  |  | 0 |  |  | 0 |  |

| Table S6: Association of pharmacodynamic and pharmacokinetic drug–drug interactions with adverse outcome events | | |
| --- | --- | --- |
|  | Pharmacodynamic Interaction | Pharmacokinetic Interaction |
| Major bleeding | 1.24 (0.84; 1.83), p=0.28 | 1.39 (0.98; 1.98), p=0.06 |
| Clinically relevant non-major bleeding | 1.25 (0.92; 1.68), p=0.15 | 0.96 (0.73; 1.28), p=0.79 |
| Any bleeding | 1.25 (0.98; 1.60), p=0.075 | 1.12 (0.89; 1.40), p=0.35 |
| Ischemic stroke/systemic embolism | 1.18 (0.69; 1.99), p=0.55 | 0.60 (0.34; 1.05), p=0.075 |
| Data are presented as Hazard Ratio (95% Confidence Intervals) | | |

Swiss-AF Investigators

University Hospital Basel and Basel University: Stefanie Aeschbacher, Steffen Blum, Leo H. Bonati, Désirée Carmine, David Conen, Ludvig Dahlheim, Urs Fischer, Corinne Girroy, Andreas Gasser, Elisa Hennings, Philipp Krisai, Michael Kühne, Vincent Meier, Christine Meyer-Zürn, Pascal B. Meyre, Andreas U. Monsch, Christian Müller, Stefan Osswald, Rebecca E. Paladini, Raffaele Peter, Adrian Schweigler, Christian Sticherling, Gian Völlmin.

Principal Investigator: Stefan Osswald; Local Principal Investigator: Michael Kühne

University Hospital Bern: Faculty: Drahomir Aujesky, Juerg Fuhrer, Laurent Roten, Simon Jung, Heinrich Mattle; Research fellows: Seraina Netzer, Luise Adam, Carole Elodie Aubert, Martin Feller, Axel Loewe, Elisavet Moutzouri, Claudio Schneider; Study nurses: Tanja Flückiger, Cindy Groen, Lukas Ehrsam, Sven Hellrigl, Alexandra Nuoffer, Damiana Rakovic, Nathalie Schwab, Rylana Wenger, Tu Hanh Zarrabi Saffari. Local Principal Investigators: Nicolas Rodondi, Tobias Reichlin

Stadtspital Triemli Zurich: Roger Dillier, Michèle Deubelbeiss, Franz Eberli, Christine Franzini, Isabel Juchli, Claudia Liedtke, Samira Murugiah, Jacqueline Nadler, Thayze Obst, Jasmin Roth, Fiona Schlomowitsch, Xiaoye Schneider, Peter Sporns, Katrin Studerus, Noreen Tynan, Dominik Weishaupt. Local Principal Investigator: Andreas Müller

Kantonspital Baden: Corinne Friedli, Silke Kuest, Karin Scheuch, Denise Hischier, Nicole Bonetti, Alexandra Grau, Jonas Villinger, Eva Laube, Philipp Baumgartner, Mark Filipovic, Marcel Frick, Giulia Montrasio, Stefanie Leuenberger, Franziska Rutz. Local Principal Investigator: Jürg-Hans Beer

Cardiocentro Lugano: Angelo Auricchio, Adriana Anesini, Cristina Camporini, Maria Luce Caputo, Rebecca Peronaci, Francois Regoli, Martina Ronchi. Local Principal Investigator: Giulio Conte

Kantonsspital St. Gallen: Roman Brenner, David Altmann, Karin Fink, Michaela Gemperle. Local Principal Investigator: Peter Ammann

Hôpital Cantonal Fribourg: Mathieu Firmann, Sandrine Foucras, Martine Rime. Local Principal Investigator: Michael Kühne

Luzerner Kantonsspital: Benjamin Berte, Kathrin Bühler, Virgina Justi, Frauke Kellner-Weldon, Richard Kobza, Melanie Koch, Brigitta Mehmann, Sonja Meier, Myriam Roth, Andrea Ruckli-Kaeppeli, Ian Russi, Kai Schmidt, Mabelle Young. Local Principal Investigator: Michael Kühne

Ente Ospedaliero Cantonale Lugano: Elia Rigamonti, Carlo Cereda, Alessandro Cianfoni, Serena Staglianò, Maria Luisa De Perna, Jane Frangi-Kultalahti, Patrizia Assunta Mayer Melchiorre, Anica Pin, Tatiana Terrot, Luisa Vicari. Local Principal Investigator: Giorgio Moschovitis.

University Hospital Geneva: Georg Ehret, Hervé Gallet, Elise Guillermet, Francois Lazeyras, Karl-Olof Lovblad, Patrick Perret, Philippe Tavel, Cheryl Teres. Local Principal Investigator: Dipen Shah

University Hospital Lausanne: Nathalie Lauriers, Marie Méan, Alessandra Pia Porretta, Sandrine Salzmann, Jürg Schläpfer. Local Principal Investigator: Michael Kühne

Bürgerspital Solothurn: Andrea Grêt, Jan Novak, Sandra Vitelli. Local Principal Investigator: Frank-Peter Stephan

Ente Ospedaliero Cantonale Bellinzona: Jane Frangi-Kultalahti, Augusto Gallino, Luisa Vicari. Local Principal Investigator: Marcello Di Valentino

University of Zurich/University Hospital Zurich: Helena Aebersold, Fabienne Foster, Matthias Schwenkglenks.

Medical Image Analysis Center AG Basel: Marco Düring, Tim Sinnecker, Anna Altermatt, Michael Amann, Petra Huber, Manuel Hürbin, Esther Ruberte, Alain Thöni, Jens Würfel, Vanessa Zuber.

Clinical Trial Unit Basel: Michael Coslovsky (Head), Pia Neuschwander, Patrick Simon, Olivia Wunderlin

Schiller AG Baar: Ramun Schmid, Christian Baumann
